# Memory Phenotype Tfh Cells Develop Without Overt Infection and Support Germinal Center Formation and B Cell Responses to Viral Infection

**DOI:** 10.1002/eji.202451291

**Published:** 2024-11-20

**Authors:** Alistair L. J. Symonds, Zabreen Busharat, Mengmeng Du, Tizong Miao, Suling Li, Xiujuan Hou, Ping Wang

**Affiliations:** ^1^ The Blizard Institute, Barts and The London School of Medicine and Dentistry Queen Mary University of London London UK; ^2^ Bioscience Brunel University London UK; ^3^ Division of Rheumatology, Dong Fang Hospital Beijing University of Chinese Medicine Beijing China

**Keywords:** CD4 T cells, Egr2, memory phenotype (MP) T cells, Tfh cells

## Abstract

Pathogen‐induced memory Tfh cells are important to maintain high‐affinity antibodies against pathogens. We have now discovered Tfh cells with a similar memory phenotype (MP) that develop in pathogen‐free conditions. These MP Tfh cells are similar to pathogen‐induced memory Tfh in both phenotype and function. They express FR4 and Egr2, which are both found in pathogen‐induced memory Tfh cells. FR4^+^Egr2^+^ CD4 MP cells express genes involved in the development of Tfh cells and homeostatic proliferation, as well as key metabolic pathways discovered in pathogen‐induced memory Tfh cells. MP Tfh cells can support B cell–mediated IgG production in vitro and induce germinal center formation and anti‐viral antibodies in response to virus infection. These mouse MP Tfh cells share a similar phenotype to human circulating Tfh cells that are increased in Sjögren's syndrome patients. Although Foxp3‐positive circulating T follicular regulatory (Tfr) cells are normal, a proportion of circulating Tfh cells from patients express increased levels of T‐bet, which is associated with high levels of inflammatory pathology. Thus, although they do not require overt infection for their development, MP Tfh cells are important for protective immune responses, and dysregulated MP Tfh responses may play a role in autoimmunity.

## Graphical abstract

In the steady state, naïve CD4 T cells can develop into memory phenotype (MP) cells. We discovered that MP T cells include a subpopulation of Tfh cells. MP Tfh cells share key markers with pathogen‐induced memory Tfh cells and can support germinal center formation and antibody responses to infection.

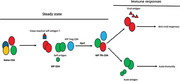

**Abbreviations**
FBS: fetal bovine serum
GF: germ‐free
MP: memory phenotype
PBMC: peripheral blood mononuclear cell
PBS: phosphate‐buffered saline
SPF: specific pathogen‐free
SS: Sjögren's syndrome
Tfr: T follicular regulatory
UMI: unique molecular identifier

## 1 Introduction

Memory phenotype (MP) T cells accumulate during aging and consist of both pathogen‐induced and virtual memory T cells, which have never encountered overt antigen stimulation [1, 2]. Although not induced by foreign antigen stimulation, virtual MP CD8 T cells, which develop under germ‐free (GF) conditions, have diverse phenotypes and both innate and adaptive functions [1, 2]. MP CD8 T cells can rapidly respond to viral infection [2, 3]. CD4 MP T cells have also been discovered and play important roles in autoimmune responses [4, 5]. Although it is unknown if MP CD4 T cells have adaptive immune functions during pathogen infection, recent studies demonstrate that about half of MP CD4 T cells express high levels of T‐bet and have innate‐like functions [6]. However, whether CD4 MP T cells have other Th functions is unknown. It has been found that circulating Tfh cells are frequently detected in the peripheral blood of healthy humans [7]. Although some of them have been found to be induced by vaccination [7], the mechanisms responsible for the generation of the remainder are unclear. Pathogen‐induced memory Tfh cells are maintained by homeostatic renewal and serve an important function in supporting memory B cells to provide long‐term antibody‐mediated protection against pathogens [8]. These memory Tfh cells carry the marker FR4 in mice, which distinguishes them from central memory T cells [8, 9]. Memory Tfh cells have a specific transcriptional profile associated with homeostatic proliferation, unique metabolic mechanisms, and Tfh function [8]. It is, however, unknown if MP Tfh cells can be generated without encountering pathogens and, if so, whether they have similar function to pathogen‐induced Tfh cells.

In this study, we found that MP Tfh cells develop in the absence of pathogen stimulation. Analysis of phenotype, molecular pathways, Tfh function, and responses against viral infection showed similar results to pathogen‐induced memory Tfh cells [8, 9]. Part of the MP Tfh cell population expresses Foxp3 and regulates Tfh cell function in induction of B cell responses. MP Tfh cells carry the FR4 marker and require the Egr2/3‐mediated transcriptional program. FR4^+^Egr2^+^ cells express genesets implicated in memory Tfh cell maintenance in the steady state [8] and support the development of germinal centers and anti‐viral antibody production in response to viral infection. We found that circulating Tfh cells in healthy donors had a similar phenotype to MP Tfh cells from mice. These cells were increased in Sjögren's syndrome (SS) patients with increased expression of T‐bet. Our data not only provide the novel discovery of MP Tfh cells but also indicate the importance of these cells in adaptive and autoimmune responses.

## 2 Results

### 2.1 MP CD4 Tfh Cells Develop in the Absence of Overt Infection

**FIGURE 1:**
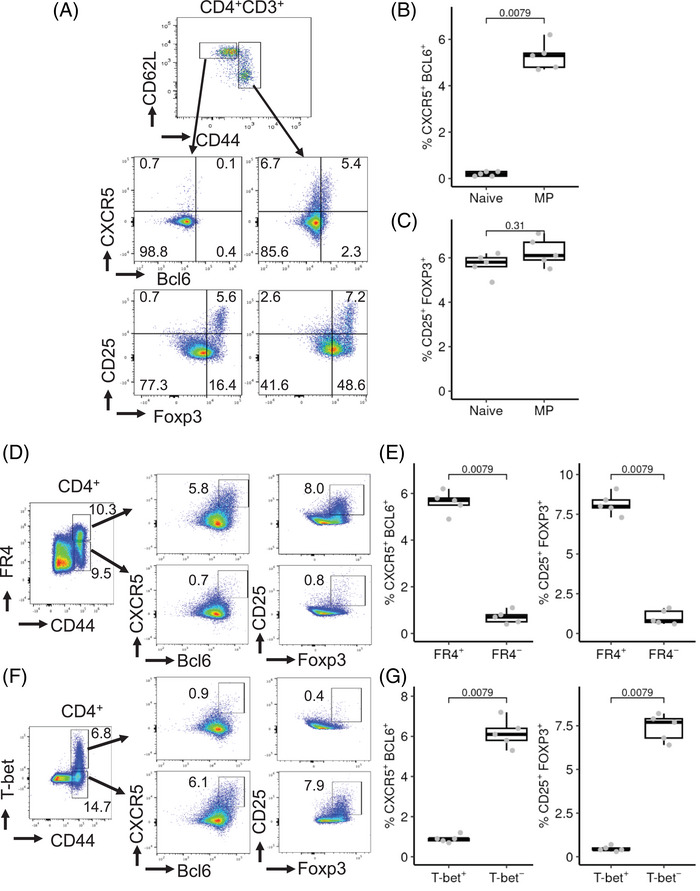
Tfh cells are present within the MP CD4 T cell population. Cells from spleen and lymph nodes of 12‐week‐old GFP‐Egr2 AmCyan‐T‐bet mice were analyzed. (A–C) CD4^+^CD62L^+^CD44^lo^ (naïve) and CD4^+^CD44^hi^ (MP) cells were gated and analyzed for Tfh (CXCR5^+^Bcl6^+^) and Treg (CD25^+^Foxp3^+^) cells. (D and E) CD4 cells were gated on the CD44^hi^FR4^+^ and CD44^hi^ FR4^−^ subpopulations for analysis of Treg and Tfh cells. (F and G) CD4 cells were gated on CD44^hi^AmCyan‐T‐bet^+^ and CD44^hi^AmCyan‐T‐bet^−^ cells and analyzed for Tfh and Treg cells. Data in (A), (D), and (F) are representative of five mice in each group and are representative of three independent experiments. In (B), (C), (E), and (G), the median, upper, and lower quartiles from five mice are shown, and data were analyzed with two‐tailed Mann–Whitney tests.

**FIGURE 2:**
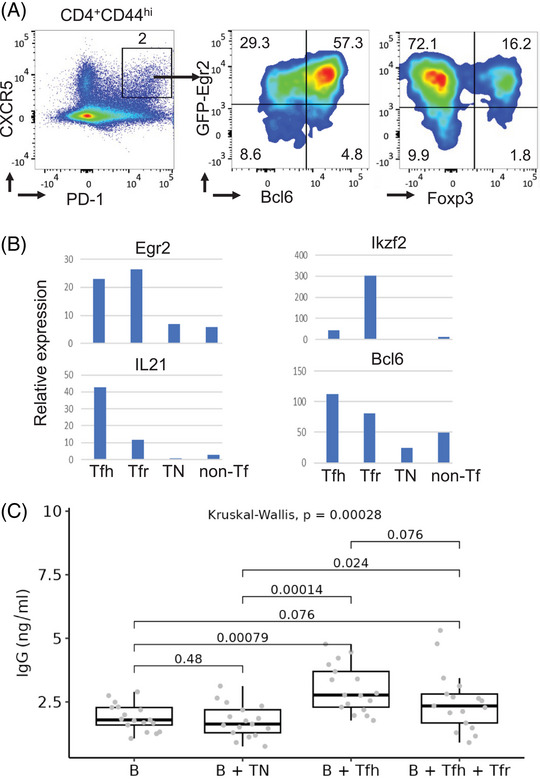
MP Tfh cells express Tfh genes and possess B helper function. (A) Cells from spleen and lymph nodes of 12‐week‐old GFP‐Egr2 AmCyan‐T‐bet mice were analyzed. Tfh (CXCR5^+^PD‐1^+^) cells among the CD4^+^CD44^hi^ MP population were gated for analysis of Bcl6, Egr2, and FoxP3. (B) CD4^+^CD44^lo^ naïve T cells (TN), CD4^+^CD44^hi^CXCR5^+^PD‐1^+^GITR^−^ MP Tfh cells, CD4^+^CD44^hi^CXCR5^+^PD‐1^+^GITR^+^ MP Tfr cells, and CD4^+^CD44^hi^CXCR5^–^PD‐1^–^GITR^−^ MP non‐follicular T cells (non‐Tf) were isolated by fluorescently activated cell sorting, and expression of the indicated genes was analyzed by real‐time RT‐PCR. (C) CD4^+^CD44^lo^ naïve T cells (TN), CD4^+^CD44^hi^CXCR5^+^PD‐1^+^GITR^–^ Tfh cells, CD4^+^CD44^hi^CXCR5^+^PD‐1^+^GITR^+^ Tfr cells, and CD4^–^B220^+^ B cells were isolated by fluorescently activated cell sorting. B cells were cultured with naïve T cells or MP Tfh cells, either alone or with MP Tfr cells, for 6 days, and IgG production was measured by ELISA. The median, upper, and lower quartiles are shown, and data were analyzed with Kruskal–Wallis tests, followed by two‐tailed Conover tests with Benjamini–Hochberg correction. Data in (A) and (B) are representative of two independent experiments, whereas that in (C) is from three independent experiments.

MP CD4 T cells can develop from naïve T cells without overt antigen stimulation [4, 5, 6]. However, it is unknown if there are Tfh cells among the pathogen‐inexperienced MP CD4 population. We analyzed the entire CD44^hi^ MP population in comparison with CD62L^+^CD44^lo^ naïve CD4 T cells from GFP‐Egr2 AmCyan‐T‐bet reporter mice bred and maintained under specific pathogen‐free (SPF) conditions. Tfh cells, characterized as CXCR5^+^Bcl6^+^, were only detected in the CD44^hi^ MP population, whereas Treg cells were present in both subsets (Figure 1A–C). Similar to antigen‐induced memory Tfh cells [8, 9], MP Tfh cells were FR4^+^ (Figure 1D,E). We found that T‐bet was highly expressed in a subset of MP CD4 T cells (Figure 1F), consistent with previous reports [6]. However, MP Tfh cells did not express T‐bet (Figure 1F,G), implying that they do not have a T‐bet‐driven innate‐like function. Some MP Tfh cells also expressed Foxp3 and so resembled T follicular regulatory (Tfr) cells (Figure 2A) [10, 11]. Our findings indicate that in the steady state, MP CD4 T cells include both Tfh and Tfr cells.

### 2.2 MP Tfh Cells Produce IL‐21 and Have B Cell Helper Function In Vitro

Memory Tfh cells that differentiate in response to viral infection can support B cell–mediated IgG production [8, 9, 10, 11, 12]. To assess the function of MP Tfh cells, MP Tfh (CD4^+^CD44^hi^PD‐1^+^CXCR5^+^GITR^−^) and MP Tfr (CD4^+^CD44^hi^PD‐1^+^CXCR5^+^GITR^+^) cells were isolated from mice under SPF conditions. GITR is highly expressed by Tfr cells and can be used to discriminate between Tfh and Tfr cells [13]. IL‐21 was highly expressed by MP Tfh cells but not by naïve CD4 cells or non‐follicular MP CD4 T cells (Figure 2B). The transcription factors Bcl6 and Egr2 are important for Tfh differentiation in response to pathogens [12, 13, 14] and were expressed highly in MP Tfh and MP Tfr cells (Figure 2A,B). Like conventional Treg cells, MP Tfr cells also expressed Ikzf2 (Figure 2B), encoding Helios, a transcription factor important for Treg function [15]. These results suggest a functional similarity to pathogen‐induced Tfh cells. To analyze the function of MP Tfh cells directly, we adapted a protocol used for analysis of isotype switching after in vivo antigen stimulation [13, 14, 15, 16]. After co‐culture with isogenic B cells in vitro, IgG antibodies were detected in cultures with MP Tfh cells but not control naïve T cells (Figure 2C). The induction was reduced when B cells and MP Tfh cells were co‐cultured with MP Tfr cells (Figure 2C). These findings indicate that MP Tfh cells have similar functions to viral induced memory Tfh cells for supporting B cell responses [8, 12].

### 2.3 The Transcription Factors Egr2/3 Are Essential for the Development of MP Tfh Cells

**FIGURE 3:**
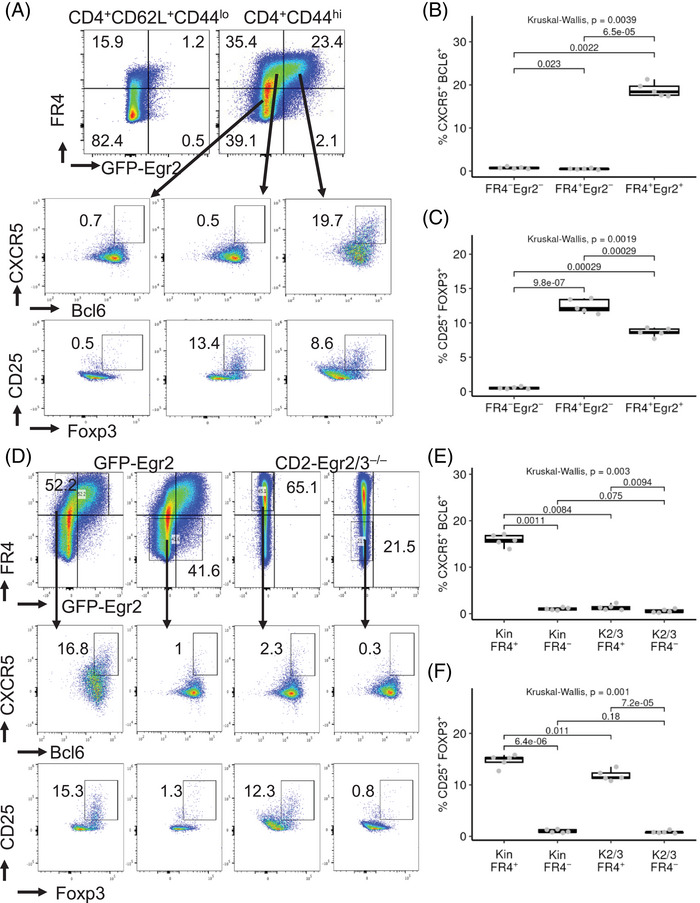
The development of MP Tfh cells is dependent on Egr2/3. (A–C) MP (CD4^+^CD44^hi^) cells from GFP‐Egr2 AmCyan‐T‐bet mice were gated on the FR4^+^Egr2^+^, FR4^+^Egr2^−^, and FR4^−^Egr2^−^ subpopulations for analysis of MP Tfh (A and B) and Treg (A and C) cells. (D–F) FR4^+^ and FR4^−^ MP CD4 cells from GFP‐Egr2 AmCyan‐T‐bet and CD2‐Egr2/3^−/−^ AmCyan‐T‐bet mice were gated for analysis of Tfh (D and E) and Treg (D and F) cells. Data in (A) and (D) are representative of five mice in each group and are representative of three independent experiments. In (B), (C), (E), and (F), the median, upper, and lower quartiles from five mice are shown, and data were analyzed with Kruskal–Wallis tests, followed by two‐tailed Conover tests with Benjamini–Hochberg correction.

The Egr2 and 3 transcription factors are important for the development of viral responding Tfh cells following viral infection by direct regulation of Bcl6 expression [14, 15, 16, 17]. Together with Bcl6, Egr2 was highly expressed in MP Tfh and MP Tfr cells (Figure 2A,B). Egr2 was also co‐expressed with FR4, a marker of memory Tfh cells [8, 9], and MP Tfh cells were only detected in the Egr2^+^ MP CD4 cell population, whereas Tregs were found in both the FR4^+^Egr2^+^ and FR4^+^Egr2^−^subsets (Figure 3A–C).

To investigate the importance of Egr2/3 in the development of MP Tfh cells further, CD2‐Egr2/3^−/−^AmCyan‐T‐bet mice, bred and maintained under SPF conditions, were used to analyze MP Tfh cells. Although the percentage of FR4^+^ MP cells in CD2‐Egr2/3^−/−^ AmCyan‐T‐bet mice was comparable to that in GFP‐Egr2 AmCyan‐T‐bet knockin mice (Figure 3D), MP Tfh cells were severely impaired in CD2‐Egr2/3^−/−^ AmCyan‐T‐bet mice (Figure 3D,E). In contrast, the proportions of CD44^hi^ Treg cells were similar in both mice (Figure 3D,F). These results indicate that the development of MP Tfh cells, but not CD44^hi^ Treg cells, requires Egr2/3.

### 2.4 Egr2/3 Regulate Genes Required for Tfh Function in MP T Cells

**FIGURE 4:**
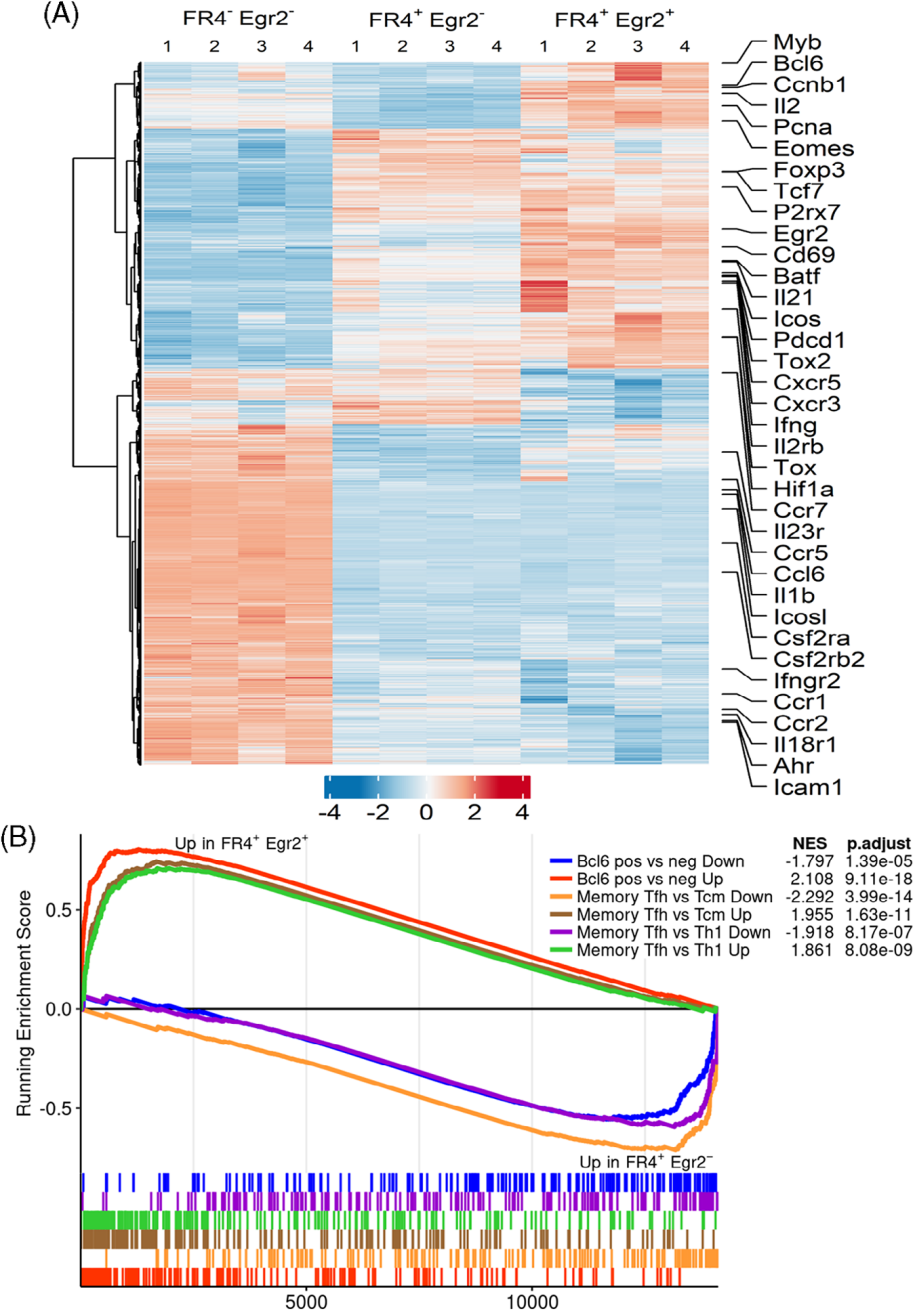
Egr2 and 3 regulate genes involved in the development of Tfh cells in MP CD4 T cells. FR4^+^Egr2^+^, FR4^+^Egr2^−^, and FR4^–^Egr2^−^ MP CD4 T cells from GFP‐Egr2 AmCyan‐T‐bet mice were analyzed by RNA‐seq. (A) Unsupervised hierarchical clustering of differentially expressed genes showing expression patterns in the three groups. (B) Geneset enrichment analysis of FR4^+^Egr2^+^ and FR4^+^Egr2^−^ MP CD4 T cells showing enrichment of Bcl6^+^ Tfh and memory Tfh cell genesets derived from Liu et al. [19] and Kunzli et al. [8], respectively. Normalized enrichment scores and Benjamini–Hochberg adjusted *p* values are shown. The RNA‐seq data are from four biological replicates, each with cells pooled from 10 mice, for each group.

Egr2/3‐regulated genes involved in the development of Tfh cells in response to viral infection [14, 15, 16, 17, 18]. MP Tfh cells expressed both FR4, a marker of memory Tfh cells [8, 9], and Egr2 (Figure 3A). Therefore, we analyzed the transcriptional profiles of FR4^+^Egr2^+^, FR4^+^Egr2^−^, and FR4^−^Egr2^−^ MP CD4 T cells. Although both FR4^+^Egr2^+^ and FR4^+^Egr2^−^ MP CD4 cells expressed memory T cell markers, such as Tcf7, P2rx7, Cxcr3, and Tox2, FR4^+^Egr2^+^ MP CD4 cells showed a distinct expression profile. FR4^+^Egr2^+^ MP CD4 T cells had high expression of genes required for homeostatic proliferation, such as Myb, Pcna, Ccnb1, and Il2, and genes expressed by Tfh cells, including Bcl6, Cxcr5, Pdcd1, and Il21 (Figure 4A). In contrast, FR4^−^Egr2^−^ MP CD4 T cells expressed central memory markers such as Ccr7 and markers of inflammation, such as Icam1, Icosl, Ahr, and Il1b (Figure 4A, Tables S1–S3). A geneset enrichment analysis of FR4^+^Egr2^+^ compared to FR4^+^Egr2^−^ MP CD4 cells revealed an enrichment of genes expressed by Bcl6^+^ Tfh cells [19] and those expressed by LCMV‐specific memory Tfh cells [8] (Figure 4B). In addition, genesets involved in growth and metabolism were enriched in FR4^+^Egr2^+^ MP cells, whereas inflammatory response genes were enriched in FR4^+^Egr2^−^ MP cells (Figure S1). It has been found that pathogen‐induced memory Tfh cells express genes involved in the mTOR, HIF‐1, and glycolytic metabolic pathways and that these pathways are essential for the maintenance of memory Tfh cells [8]. These pathways were also significantly increased in FR4^+^Egr2^+^ MP CD4 T cells compared to FR4^+^Egr2^−^ MP cells (Figure 4A, Figure S1). Collectively, these data indicate that Egr2 rather than FR4 is associated with the development of MP Tfh cells and the unique metabolic profile required for their maintenance.

**FIGURE 5:**
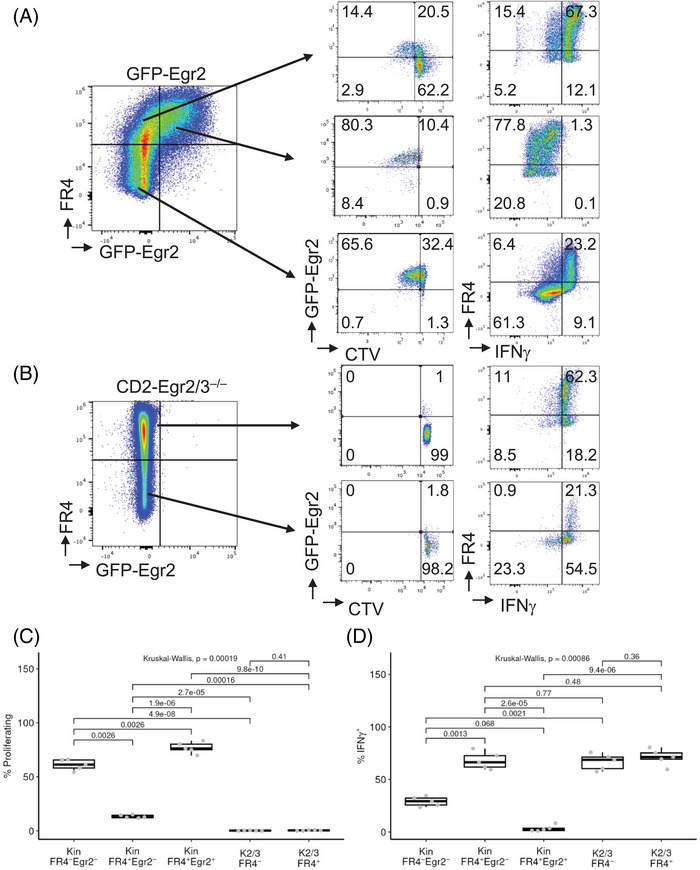
Egr2 promotes the proliferation of MP CD4 T cells in response to TCR stimulation. (A–D) FR4^+^Egr2^+^, FR4^+^Egr2^−^, and FR4^−^Egr2^−^ MP CD4 T cells were isolated from GFP‐Egr2 AmCyan‐T‐bet mice and FR4^+^ and FR4^−^ MP CD4 T cells were isolated from CD2‐Egr2/3^−/−^ AmCyan‐T‐bet mice by FACS. The isolated cells were stimulated in vitro for 72 h with anti‐CD3 and anti‐CD28 and analyzed for proliferation (A and C) and IFNγ production (B and D). Data in (A) and (B) are representative of five mice in each group and are representative of three independent experiments. In (C) and (D), the median, upper, and lower quartiles from five mice are shown, and data were analyzed with Kruskal–Wallis tests, followed by two‐tailed Conover tests with Benjamini–Hochberg correction.

To determine whether these differences in gene expression were associated with differences in function, FR4^+^Egr2^+^, FR4^+^Egr2^−^, and FR4^−^Egr2^−^ MP CD4 Tfh cells were isolated from GFP‐Egr2 AmCyan‐T‐bet mice and FR4^+^Egr2/3^−/−^ and FR4^−^Egr2/3^−/−^ MP CD4 T cells were isolated from CD2‐Egr2/3^−/−^ AmCyan‐T‐bet mice, and stimulated with anti‐CD3 and anti‐CD28 antibodies in vitro. Distinct differences in proliferation and production of IFNγ were observed. Interestingly, both the FR4^+^Egr2^+^ and FR4^−^Egr2^−^ populations proliferated more strongly than the FR4^+^Egr2^−^ subset (Figure 5A–C). Intriguingly, the proliferating cells in all subsets expressed high levels of Egr2 (Figure 5A–C), suggesting that Egr2 expression is induced in FR4^−^Egr2^−^ cells and required for supporting proliferation as demonstrated in our previous report [18]. In contrast to the poor proliferation, FR4^+^Egr2^−^ MP CD4 cells produced the most IFNγ, whereas FR4^−^Egr2^−^ MP CD4 cells produced less, and FR4^+^Egr2^+^ cells hardly produced any IFNγ (Figure 5A–D). The reciprocal differences in proliferation and production of IFNγ between FR4^+^Egr2^−^ and FR4^+^Egr2^+^ MP CD4 cells corresponded well to the differential expression of proliferation and inflammatory genes in these cell subsets (Figure 4, Figure S1, and Figure 5), suggesting that Egr2 is important in maintaining the homeostasis and controlling the inflammatory responses of MP Tfh T cells, consistent with its roles in other T cells [20]. Consistent with this notion, both FR4^+^ and FR4^−^ MP CD4 T cells from CD2‐Egr2/3^−/−^ mice hardly proliferated but produced high levels of IFNγ (Figure 5B–D).

### 2.5 MP Tfh Cells Induce Germinal Center Development and Support Neutralizing Antibody Production in Response to Viral Infection

**FIGURE 6:**
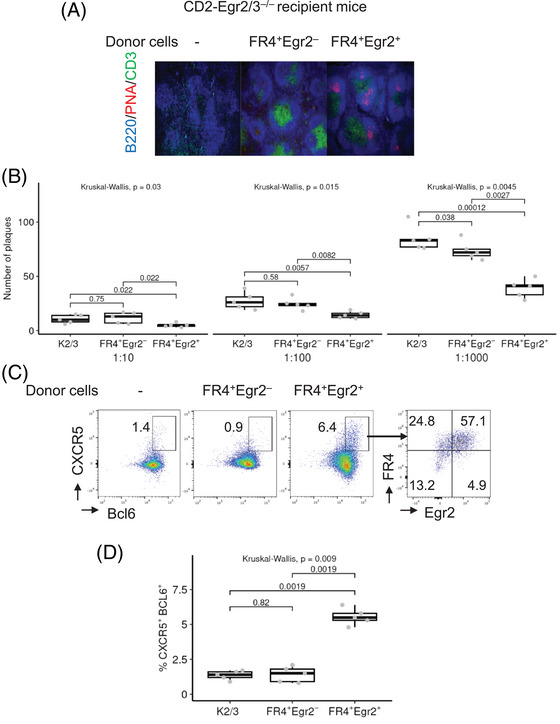
FR4^+^Egr2^+^ MP CD4 T cells restore the development of germinal centers and support B cell responses in CD2‐Egr2/3^−/−^ mice. FR4^+^Egr2^+^ and FR4^+^Egr2^−^ MP CD4 T cells were isolated from GFP‐Egr2 AmCyan‐T‐bet mice by FACS. Cells were then adoptively transferred into CD2‐Egr2/3^−/−^ mice. Twenty‐four hours after transfer, recipient mice were infected i.p. with 4 × 10^6^ PFU of vaccinia virus and analyzed 14 days later. (A) Splenic tissue sections were stained with anti‐B220 (blue), PNA (red), and anti‐CD3 (green). (B) Serum was collected from recipient mice 14 days after infection, and the presence of anti‐viral antibodies was assessed using a neutralization assay. The number of viral plaques in the presence of serum at the indicated dilutions is shown. (C and D) Tfh cells among gated CD4^+^ T cells from spleen and lymph nodes of recipient mice 14 days after infection were analyzed. Data in (A) and (C) are representative of five mice in each group and are representative of three independent experiments. In (B) and (D), the median, upper, and lower quartiles from five mice are shown, and data were analyzed with Kruskal–Wallis tests, followed by two‐tailed Conover tests with Benjamini–Hochberg correction.

Tfh cell development and function is impaired in CD2‐Egr2/3^−/−^ mice, and these mice fail to generate germinal centers in response to viral infection [14]. To test the functionality of MP Tfh cells in response to viral infection, isolated FR4^+^Egr2^−^ and FR4^+^Egr2^+^ MP CD4 cells were adoptively transferred into CD2‐Egr2/3^−/−^ mice, which were subsequently infected with vaccinia virus, and germinal center formation was analyzed. Germinal center formation was defective in CD2‐Egr2/3^−/−^ mice in response to viral infection (Figure 6A), consistent with our previous findings [14]. This defect was corrected in recipient CD2‐Egr2/3^−/−^ mice by transfer of the FR4^+^Egr2^+^ subset but not FR4^+^Egr2^−^ MP CD4 T cells (Figure 6A). The restoration of germinal center formation was associated with the development of neutralizing antibodies (Figure 6B). Tfh cells were detected in mice that received FR4^+^Egr2^+^, but not those that received FR4^+^Egr2^−^ donor cells (Figure 6C,D). Notably, the transferred FR4^+^Egr2^+^ cells expressed Egr2 in recipient mice (Figure 6C). Thus, FR4^+^Egr2^+^ MP cells can serve as functional Tfh cells in response to pathogen infection.

### 2.6 MP Tfh Cells Share Similarities With Human Circulating Tfh Cells That Are Dysregulated in Autoimmunity

**FIGURE 7:**
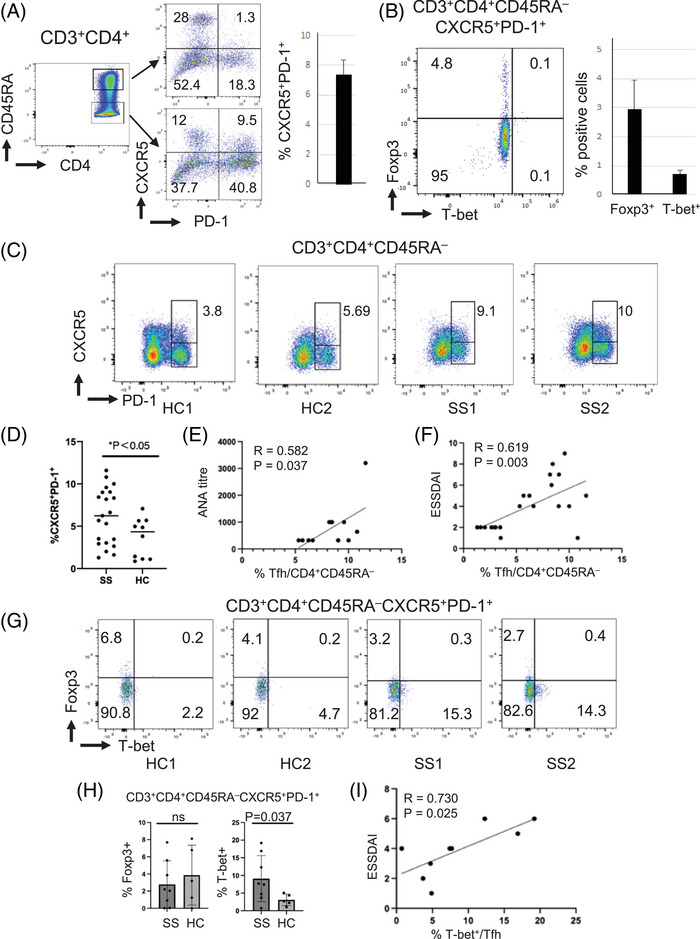
Circulating Tfh cells with a memory phenotype are increased in Sjögren's syndrome (SS) patients and contain a high percentage of T‐bet^+^ cells. (A and B) CD3^+^CD4^+^ cells from PBMCs of healthy controls were gated on CD45RA^−^ and CD45RA^+^ for analysis of CXCR5 and PD‐1 expression (A), and CXCR5^+^PD‐1^+^ cells were further analyzed for expression of Foxp3 and T‐bet (B). (C and D) CXCR5^+^PD‐1^+^ CD4 cells were compared between healthy controls and SS patients. (E and F) The association between the frequency of CXCR5^+^PD‐1^+^ cells in SS patients and the levels of anti‐nuclear antibodies (ANAs) (E) and ESSDAI (F) was analyzed. Parts (G) and (H) show the expression of Foxp3 and T‐bet in gated MP circulating Tfh cells from healthy controls and SS patients. Part (I) indicates the association of ESSDAI with the percentage of T‐bet^+^ circulating Tfh cells. Data in (A) and (B) are the mean and SD from five healthy controls and are representative of three independent experiments. Data in (D) are the mean from 21 patients and 10 controls, and data in (H) are the mean from 8 patients and 5 healthy controls, and both datasets were analyzed with Mann–Whitney two‐tailed tests. Data in (E) and (F) are from 21 patients, and data in (I) are from 8 patients, and all datasets were analyzed using Pearson correlation. N.S. not significant. *p < 0.05, **p < 0.01.

Circulating Tfh cells are frequently found in the peripheral blood of human healthy controls and are increased in autoimmune diseases [7, 21, 22, 23]. We found in healthy donors that these circulating Tfh cells all carried MP markers analogous to those expressed by mouse MP Tfh cells (Figure 7A). Although it is unknown whether these circulating MP Tfh cells are established from previous infection or developed directly from naïve CD4 T cells, the expression patterns of T‐bet and Foxp3 were similar to mouse MP Tfh cells: Some circulating Tfh cells expressed Foxp3, but circulating Tfh cells hardly expressed T‐bet (Figure 7B). These results indicate that in the steady state, Tfh and Tfr cells are part of the circulating MP T cell compartment in humans.

To assess the function of circulating Tfh cells in the development of autoimmune diseases, circulating Tfh cells (CD4^+^CD45RA^−^CXCR5^+^PD‐1^+^) from the peripheral blood of SS patients were analyzed. The frequency of circulating Tfh cells was increased in patients compared to healthy controls (Figure 7C,D). The increase was correlated with the titer of anti‐self‐antibodies and clinical manifestations (Figure 7E,F). The percentage of circulating Tfh cells expressing Foxp3 was similar between control and patient groups (Figure 7G,H). In contrast to normal levels of circulating Tfr cells, circulating Tfh cells expressing T‐bet were increased in SS patients (Figure 7G,H). The increase was associated with clinical parameters of inflammatory pathology (Figure 7I, Table S4). These results suggest that the homeostatic regulation of MP circulating Tfh cell function is important to prevent the development of autoimmune inflammatory responses in SS patients.

## 3 Discussion

MP T cells are a heterogenous population with some cells generated in response to pathogens, whereas others develop without overt antigen stimulation [1, 5, 24]. Both pathogen‐induced MP T cells and antigen‐inexperienced MP T cells are important for innate and adaptive immune responses [2, 3, 4, 5, 6, 7, 8, 9, 10, 11, 12, 13, 14, 15, 16, 17, 18, 19, 20, 21, 22, 23, 24]. We have now shown that among the MP CD4 T cells generated in the absence of infection, there is a population of MP Tfh cells. They carry the marker FR4, which has been found in pathogen‐induced memory Tfh cells [8, 9]. In addition to phenotype, MP Tfh cells have similar functions to pathogen‐induced memory Tfh cells such as facilitating B cell–mediated IgG antibody production and responses to viral infection by supporting the development of germinal centers and the production of anti‐viral antibodies. The comparable function of MP Tfh and pathogen‐induced memory Tfh cells is the result of activation of similar molecular pathways in these two types of memory Tfh cells that promote Tfh cell differentiation and homeostasis [8]. We found that circulating Tfh cells from the peripheral blood of healthy donors shared similarities to MP Tfh cells from mice. In SS patients, circulating Tfh cells were increased. Despite normal percentages of Tfr cells, some MP Tfh from patients expressed T‐bet, indicating that dysregulated MP Tfh cells may be involved in the development of autoimmunity.

Memory Tfh cells induced by pathogens are important for antibody responses during re‐infection [8]. However, in the steady state, there are antibodies that are reactive to self‐antigens [25, 26, 27]. These antibodies with moderate affinity are believed to be important for first‐line defense against pathogens [28]. Although it is unknown if MP Tfh cells are established by responses to self‐antigens, the expression of Egr2, which is induced by T cell receptor signals [18, 29, 30, 31], indicates that MP Tfh cells may be induced by self‐antigens. Indeed, the development of MP CD4 T cells requires MHC class II suggesting that self‐antigen–MHC‐mediated TCR signaling is involved in MP CD4 T cell generation [6]. The function of MP Tfh cells may be important for maintaining levels and homeostasis of low‐affinity antibodies [28]. MP Tfr cells may also be important to control these self‐reactive antibodies. The ability of MP Tfh cells to support anti‐viral antibody production indicates that these cells can not only sustain self‐reactive, low‐affinity antibodies but can also provide rapid responses to induce high‐affinity anti‐viral antibodies.

Although it is unknown how MP Tfh cells develop, we discovered that they share not only phenotypic markers, such as FR4, but also molecular signatures with pathogen‐induced memory Tfh cells [8]. MP Tfh cells not only expressed genes required for the development of Tfh cells but also expressed genes involved in metabolic pathways and homeostatic proliferation that have been identified in memory Tfh cells generated after pathogen infection [8]. Pathogen‐induced memory Tfh cells display a unique metabolic signature with high levels of genes involved in the Hif‐1, mTOR, and glycolytic pathways [8]. This metabolic state was found to be essential for maintaining memory Tfh survival [8]. FR4^+^Egr2^+^ MP CD4 cells expressed similar sets of genes indicating that the mechanisms responsible for the maintenance and function of pathogen‐induced Tfh and MP Tfh cells are similar. The differences in the requirement for exogenous antigens for their development suggest that MP Tfh cells may be cross‐reactive to different antigens in contrast to pathogen‐specific memory Tfh cells [8]. Therefore, MP Tfh cells may give rapid support to immune responses against novel pathogens. This may be particularly important in the elderly, who have a significant decline in the naïve T cell pool [32].

In the steady state, Tfr cells are critical for the suppression of autoantibody responses [33], and some evidence indicates that circulating Tfr cells are dysregulated in human autoimmune diseases [34]. We have now found that MP Tfr cells can also develop without overt infection. They can directly control the function of MP Tfh cells for induction of IgG antibody production. Interestingly, although a high level of MP‐circulating Tfh cells was found in the peripheral blood of SS patients, the percentage of Tfr cells was normal. Thus, despite the suppressive function of MP Tfr cells in vitro, it is unknown to what extent the function of these cells affects autoimmunity and immunopathology during infection.

Egr2/3 are essential for supporting proliferation of naïve T cells in responses to antigen stimulation [18, 29] and the development of Tfh cells during viral infection [14, 17]. Here we discovered that these transcription factors are also important for the development of MP Tfh cells. The genes that are regulated by Egr2/3 in MP Tfh cells include key molecules involved in Tfh cell development and function as well as those required for Tfh cell homeostasis, indicating an indispensable role of these molecules in the differentiation, maintenance, and function of Tfh and MP Tfh cells.

Circulating Tfh CD4 T cells have been reported in the peripheral blood of healthy humans, and these cells are increased in patients with autoimmune diseases [7, 21, 22, 23]. These Tfh cells share similarities with mouse MP Tfh cells: They express MP markers, and a proportion expressed Foxp3, but they did not express T‐bet (Figure 7B). Although it is difficult to define whether these are pathogen‐induced memory cells or pathogen‐independent MP cells, the regulation of the Tfh function of these cells may be important for both protective immune responses against pathogens as well as preventing auto‐reactive responses of B cells in the steady state. Indeed, MP‐circulating Tfh cells were increased in patients with SS. An increase in circulating Tfh cells has been found in most autoimmune diseases, including SS, via unknown mechanisms [23]. However, analyses of regulatory T cells among total CD4 or CXCR5^+^ cells from autoimmune patients have yielded a range of different findings [35]. Some showed an increase in Tfr cells, whereas others found either reduced numbers or similar proportions to healthy controls [36, 37]. In our cohort of SS patients, although Tfr cells were normal, T‐bet expressing MP Tfh cells were increased (Figure 7G,H). T‐bet is important for the early stage of Tfh cell differentiation in response to pathogen [38]. It is hardly expressed in circulating Tfh cells from healthy donors (Figure 7G,H), indicating that T‐bet is not required for the maintenance of circulating Tfh cells in the steady state. The inflammatory pathologies and high levels of auto‐reactive antibodies detected in these patients suggest a disorder of inflammatory Tfh function, which is yet to be further analyzed.

## 4 Data Limitations and Perspectives

Although our findings demonstrate that MP Tfh cells can be generated in the absence of overt infection, several outstanding questions remain. Prominent among them is the antigen specificity of the MP Tfh cells. MHC class II is required for the generation of all MP CD4 T cells, and, given that these cells can be generated in GF conditions, it is presumed that self‐antigen is involved [6]. However, if self‐antigen is responsible for MP Tfh cell generation, how MP Tfh cells can respond to viral antigens is still unknown. Currently, this is one of the prominent questions in the investigations of MP T cells without a clear answer. We have discussed this with one of the referees and in our discussion regarding the possibility of cross‐reactivity between self and viral antigens driving MP Tfh cell responses. We and other groups interested in MP T cells are attempting to develop models to prove or disprove this theory. If there is cross‐reactivity, the mechanism will significantly impact both anti‐pathogen responses and autoimmunity.

## 5 Concluding Remarks

MP T cells are an important part of the immune system, and their function can result in both inflammatory pathology and anti‐pathogen responses [1, 5, 24]. Our findings demonstrate that MP Tfh and regulatory MP Tfh cells are an important part of the MP CD4 T cell population. They can support humoral responses to infection and, if dysregulated, may possibly induce self‐reactive B cell responses.

## 6 Materials and Methods

### 6.1 Mice

GFP‐Egr2 AmCyan‐T‐bet and CD2‐Egr2/3^−/−^ AmCyan‐T‐bet mice were established by crossing GFP‐Egr2 [18] with AmCyan‐T‐bet mice [39], and CD2‐Egr2/3^−/−^ [29] with AmCyan‐T‐bet mice, respectively. Representative genotyping results and primer sequences are shown in Figure S2. All mouse lines are on the C57BL/6 background. All mice were bred and maintained under SPF conditions at room temperature. All mice analyzed were 10–12 weeks of age unless otherwise stated, and both male and female mice were used. No animal was excluded from the analysis, and the number of mice used was consistent with previous experiments using similar experimental designs. All mice were maintained in the Biological Services Unit, Brunel University, and protocols and procedures used were reviewed and approved by the Ethical review committee of Brunel University. Experiments were performed in accordance with UK Home Office regulations under the authority of a UK Home Office project license.

### 6.2 Antibodies and Flow Cytometry

PE‐Cy5, PerCP‐Cy5.5, APC, and eFluor450‐antibodies to CD4 (clone RM4‐5); PE‐Cy7‐anti‐IFNγ (clone XMG1.2); PE‐antibody to CD3 (clone 145‐2C11), PerCP‐efluor710‐CXCR5 (clone SPRCL5); PE, APC, eFluor450 and SuperBright780 PD‐1 (clone J43), PE, PE‐Cy7 and APC‐anti‐CD25 (clone PC61.5), PE and PE‐Cy7‐anti‐CD62L (clone MEL‐14); APC‐eFluor780 and PE‐Cy7‐antibody to CD44 (clone IM7); AlexaFluor532, PerCP‐Cy5.5, and PE‐Cy7‐anti‐FOXP3 (clone FJK‐16s) were obtained from eBioscience. AlexaFluor700‐conjugated antibodies to CD4 (clone GK1.5), APC and BV510‐anti‐CD44 antibodies (clone IM7), PE‐Cy7‐anti‐Bcl6 (clone 7D1), BV421 anti‐CXCR5 (clone L138D7), PE‐Cy7 anti‐GITR (clone DTA‐1), PE and APC/Fire750 anti‐mouse FR4 (Folate Receptor 4) antibodies (clone 12A5), and Zombie NIR were from Biolegend. PE‐labeled anti‐Bcl6 (clone K112‐91) was from BD Biosciences. For flow cytometry analysis, single‐cell suspensions were analyzed on a LSRII, Canto (BD Immunocytometry Systems) or Aurora (Cytek Biosciences), and the data were analyzed using FlowJo (Tree Star). Cell sorting was performed on a FACSAria sorter with DIVA option (BD Immunocytometry Systems).

Anti‐human antibodies: Alexa Fluor 488 Anti‐CD3 (clone UCHT1), BV786‐Anti‐CD4 (clone OKT4), BV510‐Anti‐CD45RA (clone HI100), PE‐cy7‐Anti‐PD‐1 (clone EH12.1) (also known as EH12), and BV711‐Anti‐T‐bet (clone 04–46) antibodies were obtained from BD; BV421‐Anti‐CXCR5 (clone J252D4) was from Biolegend, PE‐Anti‐Foxp3 (clone FJK‐16s) was from eBioscience. Zombie NIR Fixable Viability Kit catalog 423105 was from Biolegend.

### 6.3 Cell Isolation and Stimulation

Naïve CD4^+^ T cells were purified by negative selection using a MACS system (Miltenyi Biotec). Isolated cells were stained with anti‐CD4, anti‐CD25, and anti‐CD44. CD4^+^CD25^−^CD44^hi^FR4^+^Egr2^+^, CD4^+^CD25^−^CD44^hi^FR4^+^Egr2^−^, CD4^+^CD25^−^CD44^hi^FR4^−^Egr2^−^, CD4^+^CD25^−^CD44^hi^FR4^+^Egr2^−/−^Egr3^−/−^, and CD4^+^CD25^−^CD44^hi^FR4^−^Egr2^−/−^Egr3^−/−^ cells were isolated by fluorescently activated cell sorting. The gating strategy is shown in Figure S3. Isolated cells were labeled with CellTrace Violet (Thermo Fisher Scientific). Labeled cells were stimulated with 5 µg/mL plate‐bound anti‐CD3 (Biolegend) and 2 µg/mL anti‐CD28 (Biolegend) antibodies for 72 h before harvest. Half of the cells were harvested for analysis of proliferating cells. The other half were stimulated with 100 ng/mL ionomycin (Sigma) and 50 ng/mL PMA (Sigma) with brefeldin A (eBioscience) for 3 h. The stimulated cells were analyzed for IFNγ‐producing cells by intracellular cytokine staining.

For analysis of Bcl6 and FoxP3 expression, the cells were processed using the Foxp3 staining kit (eBioscience) and stained with anti‐Bcl6 and anti‐Foxp3 antibodies before analysis by flow cytometry.

### 6.4 In Vitro Tfh Function Assay

CD4^+^ T cells were purified by negative selection using a MACS system (Miltenyi Biotec). CD4‐negative cells were stained with anti‐CD4 and anti‐B220, whereas CD4^+^ T cells were stained with anti‐CD4, anti‐CD44, anti‐CXCR5, anti‐PD‐1, and anti‐GITR. CD4^−^B220^+^ B cells, CD4^+^CD44^lo^ naïve T cells, CD4^+^CD44^hi^CXCR5^+^PD‐1^+^GITR^−^ Tfh cells, and CD4^+^CD44^hi^CXCR5^+^PD‐1^+^GITR^+^ Tfr cells were isolated by fluorescent activated cell sorting. The gating strategies are shown in Figure S3. 1 × 10^5^ B cells were cultured with 3 × 10^4^ Tfh cells or naïve T cells in the presence of 2 µg/mL anti‐CD3 and 5 µg/mL anti‐IgM. 1.5 × 10^4^ Tfr cells were added where indicated. Supernatants were collected 6 days later, and total IgG production was analyzed by ELISA using a Total IgG Mouse ELISA Kit (Invitrogen) according to the manufacturer's instructions. Absorbance at 450 and 570 nm was measured using a CLARIOstar plate reader (BMG Labtech). Absorbance at 570 nm was subtracted from the 450 nm readings, and 4 and 5 parameter weighted and unweighted models were fitted to the standard curve data. Unweighted 4 parameter models proved the best fit for the data and were used to calculate the amounts of IgG within the samples.

### 6.5 Quantitative Real‐Time PCR

Total RNA was extracted from cells using the RNeasy UCP Micro Kit (Qiagen) and reverse transcribed using SuperScript IV Reverse Transcriptase with random primers (Invitrogen). Quantitative real‐time PCR was performed on a Rotor‐Gene system (Corbett Robotics) using SYBR green PCR master mix (Qiagen). The primers used were as follows: Egr2: sense 5′‐CTTCAGCCGAAGTGACCACC‐3′ and antisense 5′‐GCTCTTCCGTTCCTTCTGCC‐3′; Bcl6: sense 5’‐CATGCAGGAAGTTCATCAAGG‐3’ and antisense 5’‐CTCAGTGGCATATTGTTCTCC‐3’; Il21: sense 5’‐CTCAAGCCATCAAACCCTGG‐3’ and antisense 5’‐CATACGAATCACAGGAAGGG‐3’; Ikzf2: sense 5’‐AACGCTGTCACAACTATCTCC‐3’ and antisense 5’‐CTTTCCCATATTTGCCGTGAG‐3’; and β‐actin sense 5′‐AATCGTGCGTGACATCAAAG‐3′ and antisense 5′‐ATGCCACAGGATTCCATACC‐3′.

The data were analyzed using the Rotor‐Gene Software. All samples were run in triplicate, and relative mRNA expression levels were obtained by normalizing against the level of β‐actin from the same sample under the same program using: relative expression = 2^(CTβ‐actin − CTtarget).

### 6.6 RNA‐Seq Analysis

RNA was isolated and purified using the RNeasy UCP Micro Kit (Qiagen) according to the manufacturer's instructions. RNA concentration and integrity were assessed using Qubit with an RNA HS reagent kit (Thermo Scientific) and an Agilent 4200 Tapestation 2100 Bioanalyzer (Agilent Technologies), respectively. mRNA from independent biological replicates was processed for mRNA‐Seq library construction using the Kapa mRNA HyperPrep Kit (Roche) in combination with IDT xGen UDI‐UMI adapters (Integrated DNA Technologies) according to the manufacturers’ protocols. Single‐end sequencing of 75 bp was performed using an Illumina NextSeq 500 platform, along with an 8 bp index and 8 bp of the unique molecular identifiers (UMIs). Run data were demultiplexed and converted to fastq files using Illumina's bcl2fastq Conversion Software v2.20. The sequenced reads were mapped to the mm10 build of the mouse reference genome using the spliced aligner Hisat2 version 2.2.0 [40]. Intermediate processing steps to remove secondary alignments were performed using SAMtools version 1.10 [41], whereas duplicates were removed using the UMI information with UMI‐tools version 1.1.2 [42]. The number of reads mapped to each gene based on the UCSC mm10 version of the NCBI RefSeq database was then quantified using the featureCounts function in the Rsubread package version 2.4.3 [43] of the R programming language version 4.0.2 [44]. Further steps were then conducted using the R programming language version 4.0.5 [44]. Count data were then normalized and dispersion estimated before a negative binomial model was fitted with significance assessed by a quasi‐likelihood F‐test using the package edgeR version 3.32.1 [45, 46]. Genes with an adjusted *p* value less than or equal to 0.05 and an absolute fold change greater than or equal to 1.5 were considered differentially expressed.

For the heatmap, a variance stabilizing transformation from the DESeq2 and vsn packages, versions 1.30.1 and 3.58.0, respectively [47, 48], was applied to the dataset and *Z* scores were calculated for each gene before clustering using 1 – Pearson correlation as a distance metric and visualization of differentially expressed genes with the ComplexHeatmap package version 2.6.2 [49].

For functional annotation, the msigdbr package version 7.5.1 [50] was used to obtain Mouse versions of the Hallmark and Immunological signature MSigDB genesets [51]. The immunological signature genesets were supplemented by the addition of genesets generated from memory Tfh cells described in Kunzli et al. [8]. The filtered UMI matrix was downloaded from GEO (accession number GSE134157), and the data were processed using the methods described in Kunzli et al. [8], as far as possible. Briefly, the data were normalized using the deconvolution method in the R (version 4.3.2) package scran (version 1.30.2) [52], and the top 500 genes by biological variance were used to perform PCA, which was then denoised to remove modeled technical variation. Clusters were determined using a graph‐based method with shared neighbor weighting, which identified seven clusters as in Kunzli et al. [8]. The clusters with the highest expression of Izumo1r, encoding FR4, Pdcd1, encoding PD‐1, and Cxcr5 were considered to be memory Tfh cells, whereas the two clusters with the highest expression of Ccl5 and Cxcr6 were considered to be memory Th1 cells. Two of the remaining clusters had high levels of Itgb1 and Il7r but low levels of Izumo1r, so they were considered to be Tcm cells. The remaining cluster expressed both Tfh and Tcm markers and was excluded from further analysis. Pseudobulk samples were generated from the memory Tfh, memory Th1, and Tcm clusters identified above and differentially expressed genes identified using edgeR version 4.0.16 [45, 46]. The top 200 differentially expressed genes for each comparison were then added to the immunological signature genesets for GSEA. Pre‐ranked GSEA was performed using the clusterProlifer and fgsea packages versions 3.18.1 and 1.16.0, respectively [53, 54], using the quasi‐likelihood *F*‐statistic multiplied by the sign of the log2 fold change as the ranking metric. In Figure 4B, the “Bcl6 pos vs neg Up” and “Bcl6 pos vs neg Down” genesets are GSE40068_BCL6_POS_VS_NEG_CXCR5_POS_TFH_UP and GSE40068_BCL6_POS_VS_NEG_CXCR5_POS_TFH_DOWN, respectively (both from Liu et al [19]), whereas the remaining genesets are those generated from the data in Kunzli et al. [8] as described above.

For the volcano plot, the Benjamini–Hochberg‐corrected *p* values and log2 fold changes, calculated from the edgeR data, for the total dataset were plotted using the ggplot2 package version 3.3.6 [55], and then selected genes were highlighted.

### 6.7 Adoptive Transfer

CD4^+^CD25^−^CD44^hi^FR4^+^Egr2^+^ and CD4^+^CD25^−^CD44^hi^FR4^+^Egr2^−^ cells were isolated from GFP‐Egr2 AmCyan‐T‐bet mice by fluorescence‐activated cell sorting. 2 × 10^5^ cells from each subset were suspended in 100 µL of physiological saline and injected i.v. into the dorsal tail vein of 6‐ to 8‐week‐old CD2‐Egr2/3^−/−^ mice. Twenty‐four hours after transfer, the recipient mice were infected i.p. with 4 × 10^6^ PFU of vaccinia virus. Fourteen days after infection, Tfh cells, neutralizing antibody titers, and GC formation were analyzed.

### 6.8 Viruses

VVWR stocks were grown using TK143 cells in T175 flasks, infected at a multiplicity of infection of 0.5. Cells were harvested at 72 h, and virus was isolated by rapidly freeze‐thawing the cell pellet three times in 5 mL DMEM containing 10% fetal bovine serum (FBS) as previously described [14]. Cell debris was removed by centrifugation. Clarified supernatant was frozen at −80°C as virus stock. VVWR stocks were titrated using TK143 cells.

### 6.9 Viral Infection

Mice were infected intraperitoneally with 4 × 10^6^ PFU of vaccinia virus in 100 µL of physiological saline. The mice were weighed and observed for illness daily, as previously described [18].

### 6.10 Plaque Reduction Neutralization Tests

TK143 cells were seeded into 24‐well Costar plates (Corning Inc., Corning, NY) and used within 2 days of reaching confluence. Sera were diluted in DMEM medium. The serially diluted sera were then incubated with an equal volume of VVWR (2 × 10^4^ PFU/mL) overnight at 37°C. The cells were rinsed in serum‐free medium, the medium was aspirated, and 100 µL of virus–serum mixture was added to each well in duplicate and left to adsorb for 60 min at 37°C with periodic swirling. The wells were then washed with serum‐free medium, and normal growth medium was added. After allowing 2 days for the plaques to develop, the cells were fixed and stained in one step with 0.1% crystal violet in 20% ethanol, and the plaques were quantified over white‐light transillumination.

### 6.11 Immunohistochemistry Analysis

Spleen tissue sections were fixed with 4% paraformaldehyde in phosphate‐buffered saline (PBS) and embedded in paraffin. Paraffin sections of spleen tissues were stained using Rat anti‐mouse B220 (BD Biosciences), which was detected using anti‐Rat Far‐red‐labeled IgG (Sigma Chemical Co., St. Louis, MO, USA), and Rabbit anti‐CD3 (DAKO), which was detected using anti‐Rabbit Alexa Fluor 594‐labeled IgG (Invitrogen, Oregon, USA), together with Tritc‐conjugated PNA (Sigma). Stained sections were washed in PBS and mounted using Vectashield (Vector Laboratories, Burlingame, CA, USA).

### 6.12 Human Study

Research involving human subjects abided by the Declaration of Helsinki principles and was performed according to the guidelines from the Local Ethical Review Committee, Dong Fang Hospital, Beijing Chinese Medicine University, through approved protocols with appropriate informed consent obtained. Patients with SS fulfilled the ACR/EULAR 2016 Primary Sjögren's syndrome classification criteria. Patient characteristics were obtained by review of digital medical records (Table S4). All blood samples were obtained from SS patients seen at the Dong Fang Hospital Sjögren's Syndrome Center, Dong Fang Hospital, Beijing Chinese Medicine University. Blood samples were acquired before initiation of a new biological therapy or within 1 week of starting treatment with anti‐inflammatory agents. Peripheral blood mononuclear cells (PBMCs) were isolated from blood using Ficol (Sigma) according to the manufacturer's protocol. All blood CD4^+^ T cell analyses focused on CD45A^−^ memory (MP) CD4 T cells, which includes both resting and activated MP cells. The non‐inflammatory healthy controls were staff at Dong Fang Hospital, Beijing Chinese Medicine University.

### 6.13 Statistics

To analyze the statistical significance of differences between groups, non‐parametric tests were employed due to small sample sizes. Two‐tailed Mann–Whitney tests using the R package coin [56], or Kruskal–Wallis tests followed by pairwise comparisons using Conover tests, as implemented in the R package PMCMRplus [57], with Benjamini–Hochberg correction for multiple comparisons were used as indicated. Sample sizes and replication details are shown in the respective figure legends. No data were excluded from the analysis. Differences with a *p* value <0.05 were considered significant.

## Author Contributions

**A.L.J.S**., **T.M**., **S.L**., **P.W**., and **X.H**.: conceptualization. **Z.B**., **A.L.J.S**., and **T.M**.: methodology. **Z.B**., **A.L.J.S**., **T.M**., **M.D**., **S.L**., **P.W**., and **X.H**.: investigation. **P.W**.: writing–original draft. **P.W**., **A.L.J.S**., **T.M**., and **S.L**.: writing–review and editing. **P.W**.: writing–original draft. **P.W**., **A.L.J.S**., **T.M**., and **S.L**.: writing–review and editing. **A.L.J.S**., **T.M**., and **S.L**.: formal analysis. **P.W**. and **S.L**.: funding acquisition. **S.L**., **T.M**., and **X.H**.: resources. **P.W**., **S.L**., and **X.H**.: supervision.

## Ethics Statement

All protocols and procedures used for mouse experiments were reviewed and approved by the ethical review committee of Brunel University under the authority of a UK Home Office project license.

## Consent

Research involving human subjects was performed according to the guidelines from the Local Ethical Review Committee, Dong Fang Hospital, Beijing Chinese Medicine University, through approved protocols with appropriate informed consent obtained.

## Conflicts of Interest

The authors declare no conflicts of interest.

### Peer review

The peer review history for this article is available at https://publons.com/publon/10.1002/eji.202451291.

## Supporting information



Supporting information

Supporting information

Supporting information

Supporting information

Supporting information

## Data Availability

The RNA‐seq data generated in this study are available from the BioStudies website (www.ebi.ac.uk/biostudies) under accession number: E‐MTAB‐12736.
