# Supplementary material for: Memory Phenotype Tfh Cells Develop Without Overt Infection and Support Germinal Center Formation and B Cell Responses to Viral Infection
Source: Eur J Immunol. 2024 Nov 20;55(1):e202451291. doi: 10.1002/eji.202451291 (PMC11739680; doi:10.1002/eji.202451291)
Supplement: Supplementary file 1 — Supporting information [file EJI-55-e202451291-s002.pdf]

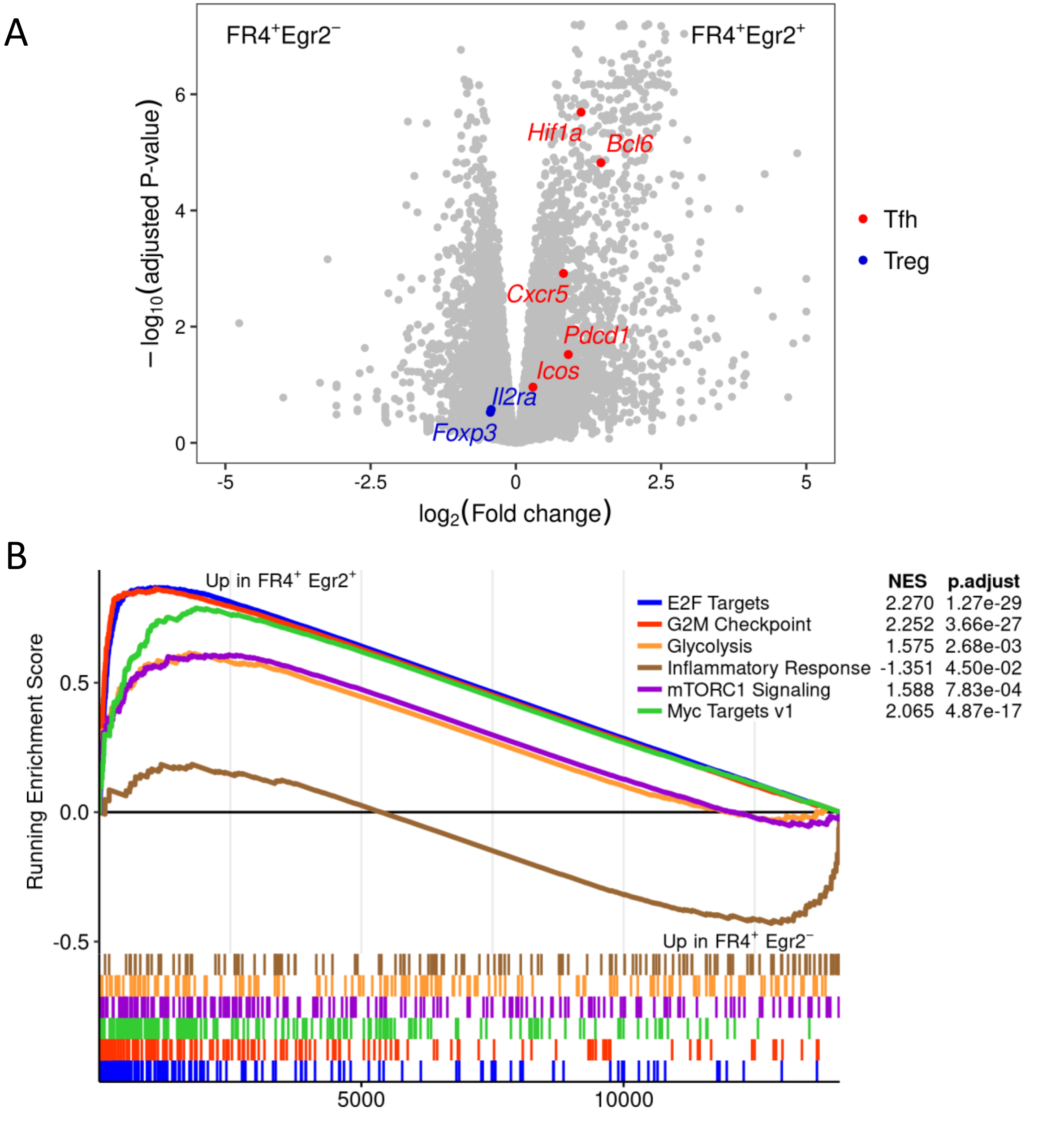

Figure S1. FR4+Egr2+ cells express Tfh genes and genesets associated with proliferation and metabolism. FR4+Egr2+, FR4+Egr2- and FR4-Egr2- MP CD4 T cells from GFP-Egr2 AmCyan-T-bet mice were analyzed by RNA-seq. A. Treg and Tfh genes in FR4+Egr2+ and FR4+Egr2- MP CD4 T cells. B. Gene set enrichment analysis of FR4+Egr2+ and FR4+Egr2- MP CD4 T cells using genesets from the Hallmark MSIGDB collection v7.5.1 (40). Normalised enrichment scores and Benjamini-Hochberg adjusted p-values are shown. The RNA-seq data are from four biological replicates, each with cells pooled from 10 mice, for each group.

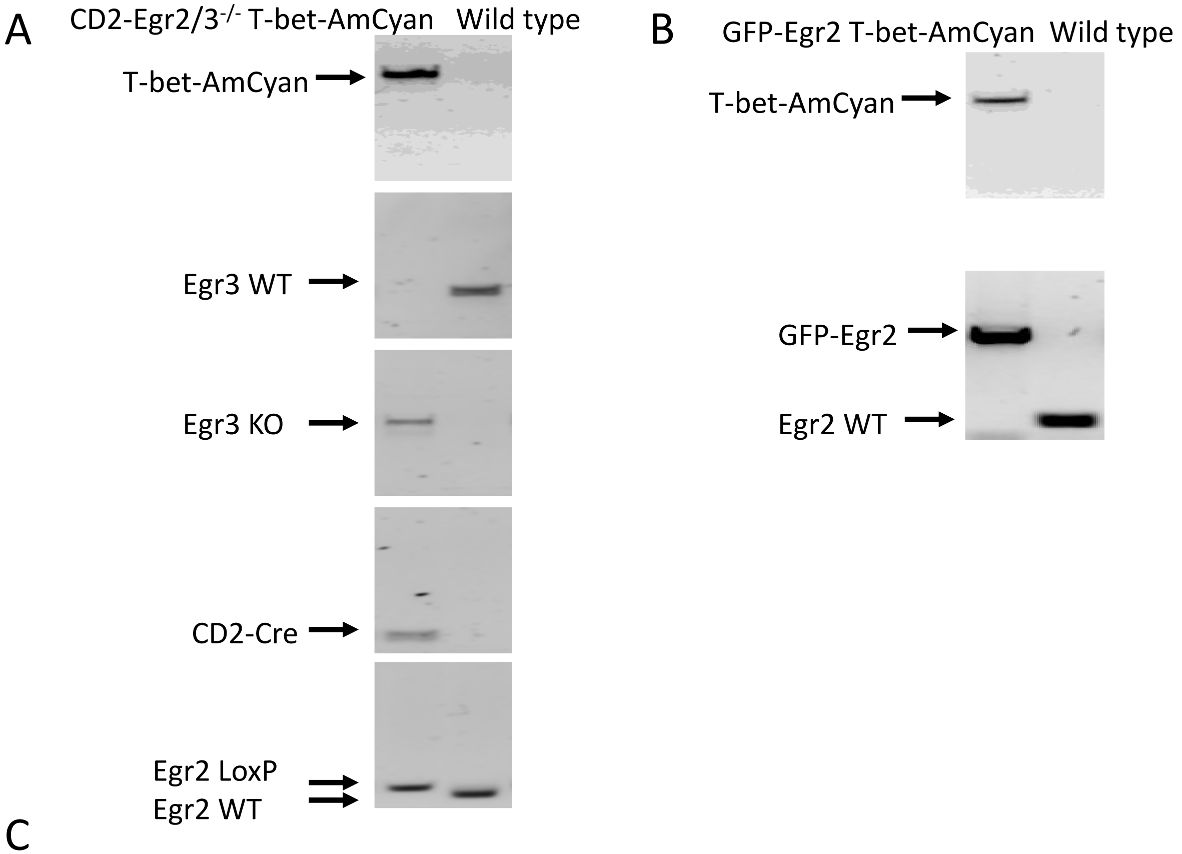

C

LOCUS	PRIMER	BAND SIZE (bp)
Egr2 LoxP	Sense – TCA GCA TGC GTG TAT GTG<br>Antisense – GAA GCT ACT CGG ATA CGG	WT = 145bp<br>LoxP = 179bp
CD2-Cre	Sense – CCA ACA ACT ACC TGT TCT GCC G<br>Antisense – TCA TCC TTG GCA CCA TAG ATC AGG	133bp
Egr3	Sense – CTA TTC CCC CCA GGA TTA CC<br>WT Antisense – TCT GAG CGG GCT GAA ACG<br>KO Antisense – GAT TGT CTG TTG TGC CCA GTC	WT = 360bp<br>KO = ~700bp
GFP-Egr2	Sense – GCT CAG TTC AAC CCC TCT CC<br>Antisense – GGA TTT TGT CTA CGG CCT TG	WT = 119bp<br>GFP-Egr2 = 854bp
T-bet-AmCyan	Sense – GAC AAG AGA CTT ACA CTT AGG AGT G<br>Antisense – GTA GGT GAA GGT TCT CTC GTA G	~600bp

Figure S2. Genotyping of CD2-Egr2/3$^{-/-}$ AmCyan-T-bet and GFP-Egr2 AmCyan-T-bet mice. DNA from tail tissues was analyzed by PCR to identify CD2-Egr2/3$^{-/-}$ AmCyan-T-bet (A) and GFP-Egr2 AmCyan-T-bet (B) mice. C. Primers for each locus and size of PCR products.

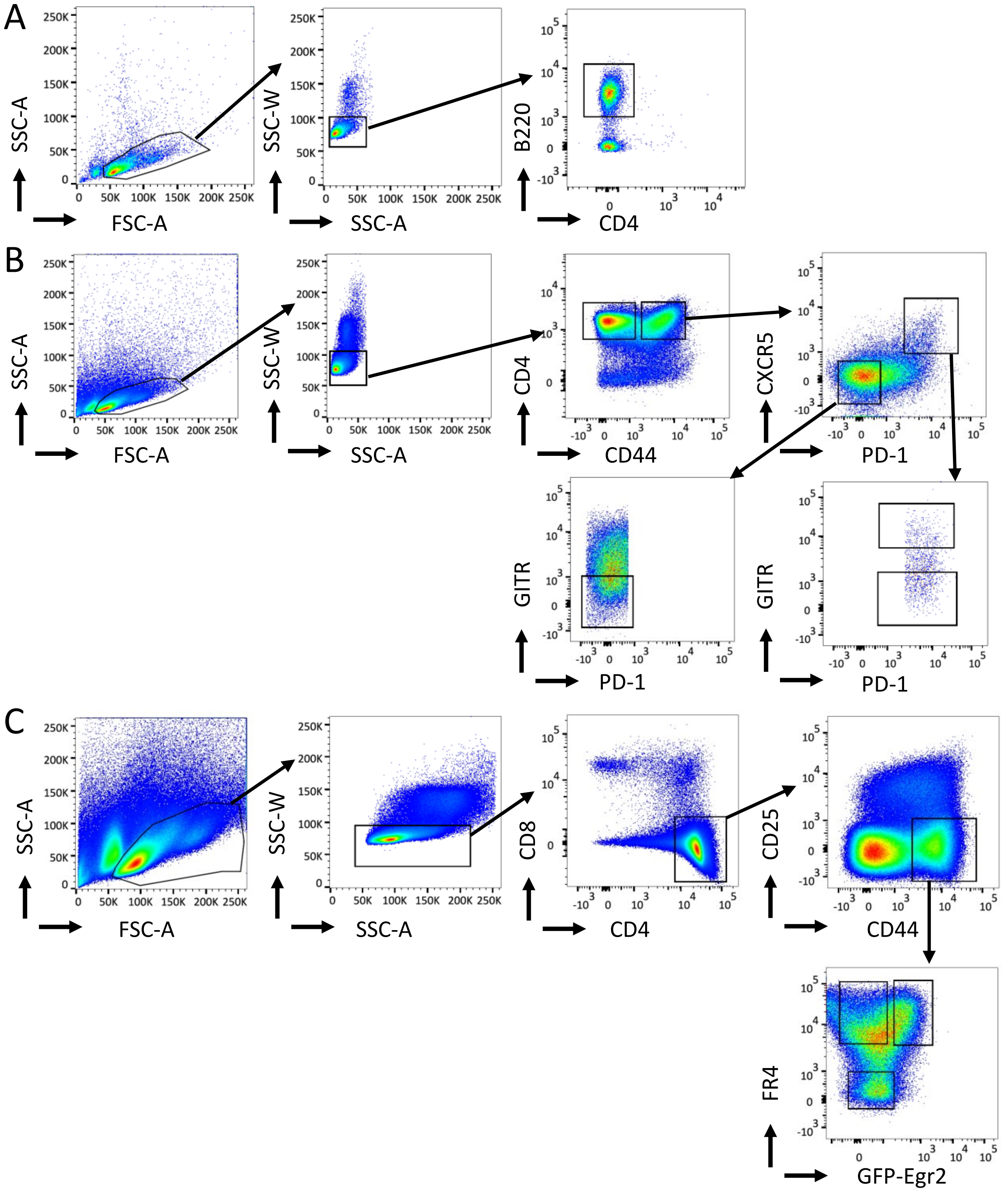

Figure S3. Gating strategies for cell sorting. A. B cell sorting strategy. CD4- cells were isolated by MACS and then B cells were isolated by FACS. B. Tfh, Tfr, non-Tf and naïve T cell sorting strategy. CD4+ cells were isolated by MACS and then Tfh, Tfr, non-Tf and naïve T cells were isolated by FACS. C. FR4+Egr2+, FR4+Egr2- and FR4-Egr2- cells were isolated by FACS.
